# Exposing Manipulated Photos and Videos in Digital Forensics Analysis

**DOI:** 10.3390/jimaging7070102

**Published:** 2021-06-24

**Authors:** Sara Ferreira, Mário Antunes, Manuel E. Correia

**Affiliations:** 1 Department of Computer Science, Faculty of Sciences, University of Porto, 4169-007 Porto, Portugal; mdcorrei@fc.up.pt; 2 Computer Science and Communication Research Centre (CIIC), School of Technology and Management, Polytechnic of Leiria, 2411-901 Leiria, Portugal; 3 INESC TEC, CRACS, 4200-465 Porto, Portugal

**Keywords:** digital forensics, cybersecurity, multimedia content manipulation, deepfake, convolutional neural networks, support vector machines, discrete fourier transform

## Abstract

Tampered multimedia content is being increasingly used in a broad range of cybercrime activities. The spread of fake news, misinformation, digital kidnapping, and ransomware-related crimes are amongst the most recurrent crimes in which manipulated digital photos and videos are the perpetrating and disseminating medium. Criminal investigation has been challenged in applying machine learning techniques to automatically distinguish between fake and genuine seized photos and videos. Despite the pertinent need for manual validation, easy-to-use platforms for digital forensics are essential to automate and facilitate the detection of tampered content and to help criminal investigators with their work. This paper presents a machine learning Support Vector Machines (SVM) based method to distinguish between genuine and fake multimedia files, namely digital photos and videos, which may indicate the presence of deepfake content. The method was implemented in Python and integrated as new modules in the widely used digital forensics application Autopsy. The implemented approach extracts a set of simple features resulting from the application of a Discrete Fourier Transform (DFT) to digital photos and video frames. The model was evaluated with a large dataset of classified multimedia files containing both legitimate and fake photos and frames extracted from videos. Regarding deepfake detection in videos, the Celeb-DFv1 dataset was used, featuring 590 original videos collected from YouTube, and covering different subjects. The results obtained with the 5-fold cross-validation outperformed those SVM-based methods documented in the literature, by achieving an average F1-score of 99.53%, 79.55%, and 89.10%, respectively for photos, videos, and a mixture of both types of content. A benchmark with state-of-the-art methods was also done, by comparing the proposed SVM method with deep learning approaches, namely Convolutional Neural Networks (CNN). Despite CNN having outperformed the proposed DFT-SVM compound method, the competitiveness of the results attained by DFT-SVM and the substantially reduced processing time make it appropriate to be implemented and embedded into Autopsy modules, by predicting the level of fakeness calculated for each analyzed multimedia file.

## 1. Introduction

Cybercrime has challenged national security systems all over the world, and, in the last five years, there has been an increase of 67% in the incidence of security breaches worldwide [1], with malicious activities like phishing, ransomware, and cryptojacking being the most popular threats to cybersecurity [2,3,4]. In a broad sense, malicious actors are taking advantage of human and technical vulnerabilities, to steal and acquire illicit benefits from victims. The widespread global reach of cyberattacks, their level of impact, sophistication, and dire consequences for society can be analyzed within several distinct dimensions, namely economic disruption, psychological disorder, and other threats to national defense [5,6]. The pandemic we are all currently enduring has also raised a global awareness about how dependent we now are on the Internet to carry on a semblance of normal life. Activities we took for granted in our daily lives, like working, reading, talking, studying, and shopping, are now highly dependent on digital services. This creates a perfect context for an increase in online fraud and other criminal activities in cyberspace [7,8].

Defacing and deepfakes take advantage of multimedia content manipulation techniques to tamper digital photos and videos. They can inflict severe reputational and other kinds of damages on their victims. These cyberthreats use hyper-realistic videos that apply Artificial Intelligence (AI) techniques to change what someone says and does [9]. Coupled with the reach and speed of social media, convincing deepfakes can quickly reach millions of people, negatively impact society in general and create real havoc on the lives of its victims. A deepfake attack may have different motivations. Fake news [10], revenge porn [11], and digital kidnapping, usually involving under-aged or otherwise vulnerable victims [12], associated with ransomware blackmailing, are among the most relevant forms of deepfaking attacks that can create havoc on the lives of its victims.

Digital forensics analysis is carried out mainly by the criminal investigation police. It embodies techniques and procedures for the collection, preservation, and analysis of digital evidence that may exist in electronic equipment [13]. Digital forensics techniques are essential to investigating crimes that are committed with computers (e.g., phishing and bank fraud), as well as those carried on against individuals, where evidence may reside on a computer (e.g., money laundering and child exploitation) [14].

When conducted manually, solely by the means of a human operator, digital forensics can be very time-consuming and highly inefficient in terms of identifying and collecting complete and meaningful digital evidence of cybercrimes [15]—in a process akin to the proverbial “search of a needle in a Haystack”. Moreover, the manual analysis of multimedia content, for the identification of manipulated videos or photos, often results in the misclassification of files.

Effective forensic tools are essential, as they have the ability to reconstruct evidence left by cybercriminals when they perpetrate a cyberattack [16]. However, there exists an increasing number of highly sophisticated tools that make life much easier for cyber-criminals to carry out complex and highly effective digital attacks. The criminal investigator is thus faced with a very difficult challenge in trying to keep up with these cyber-criminal operational advantages [17]. Autopsy (https://www.autopsy.com/ (accessed on 22 June 2021)) is a digital forensic tool that helps to level out this field. It is open-source and widely used by criminal investigators to analyze and identify digital evidence and artifacts of suspicious and anomalous activities. It incorporates a wide range of native modules to process digital objects, including images (on raw disks), and also provides a highly effective framework that allows the community to develop more modules for otherwise more specialized forensic tasks.

Machine Learning (ML) has boosted the automated detection and classification of digital artifacts for forensics investigative tools. Existing ML techniques to detect manipulated photos and videos [18] are seldom not fully integrated into forensic applications. Therefore, ML-based Autopsy modules, capable of detecting deepfakes are relevant and will most certainly be very much appreciated by the investigative authorities. The good results already observed by the reported ML methods for deepfake detection have not yet been fully translated into substantial gains for cybercrime investigation, as those methods have not often been incorporated into the most popular state-of-the-art digital forensics tools [19].

This paper describes the deployment and development of a standalone application to detect both digital photos as well as videos that have been manipulated and may be part of a deepfake attack. The application was further deployed as two separate modules for Autopsy, namely one to detect manipulated digital photos and other manipulated videos. The standalone application and the Python modules developed for Autopsy incorporate an SVM-based model [20] to detect discrepancies in photos and video frames, namely splicing and copy–move anomalies. It works by extracting a set of fifty features calculated by a Discrete Fourier Transform (DFT), applied to the input files that are then further processed by an SVM-based method. These Autopsy modules were tested with a classified dataset of about 40,000 photos and 800 videos, composed of both faces and objects, where it is possible to find examples of slicing and copy–move manipulations. One part of this dataset consists of frames from deepfake videos that are part of the Celeb-DFv1 dataset [21]. The results obtained prove that Support Vector Machines (SVM) -based methods can attain very good precision on the detection of both tampered photos and videos. Regarding photos, we have achieved a mean precision of 99.6% and a F1-score of 99.5%, with a 5-fold cross-validation. Manipulated video detection achieved a mean precision of 74.4% and a F1-score of 79.5%. When processing photos and videos altogether, a mean precision of 81.1% and an F1-score of 87.9% were obtained.

The contributions of this paper can be outlined as follows:

- A labeled dataset composed of about 40,000 multimedia files. It incorporates state-of-the-art datasets of both normal examples and those subjected to some kind of manipulation, namely splicing, copy–move and deepfaking.
- An SVM-based model to process multimedia files and to detect those that were digitally manipulated. The model processes a set of simple features extracted by applying a DFT method to the input file. The tests were performed on the newly created dataset.
- The development of two ready-to-use Autopsy modules. One to detect the fakeness level of digital photos and the other to detect the fakeness level of input video files. The Autopsy modules take advantage of the SVM-based model implemented as a standalone application and have been made available in the following GitHub repository: https://github.com/saraferreirascf/Photo-and-video-manipulations-detector (accessed on 22 June 2021). The datasets are also available in the GitHub link, and the modules are ready to be installed and used in Autopsy.

The remainder of this paper is organized as follows. Section 2 describes the most up-to-date methods used to detect multimedia content manipulation, followed by a comprehensive description of the main fundamentals and methods behind the subject of deepfake detection. Section 3 explains digital forensics and characterizes some key concepts behind the Autopsy forensics tool, namely the set of existing available ingest modules. Section 4 depicts the overall architecture and the multimedia files process pipeline, delineating the overall benchmark process of the deepfake multimedia dataset. The experimental setup environment and the datasets processed by our experiments are described in Section 5. Section 6 presents the performance metrics used, the results obtained followed by their corresponding analysis. Finally, Section 7 states the main conclusions and delineates some future work.

## 2. Literature Review and State of the Art

This section describes some of the fundamentals behind digital forensics and multimedia manipulation techniques. It also surveys some of the most relevant and popular ML techniques that can be used to detect fake multimedia content.

### 2.1. Multimedia Manipulation Techniques

Digital photos and video manipulation is a very appealing and highly effective medium to spread misinformation. There are three popular main types of manipulation that can be applied to a multimedia file, namely copy–move, splicing, and deepfake. Despite the similarity of the overall final result, as it consists mainly of manipulating objects, faces, or voices in multimedia files, the methods to produce and further enhance the manipulation and then the ML techniques employed to detect these manipulations are very distinct and can be highly challenging for a fully automated detection system.

**Figure 1:**
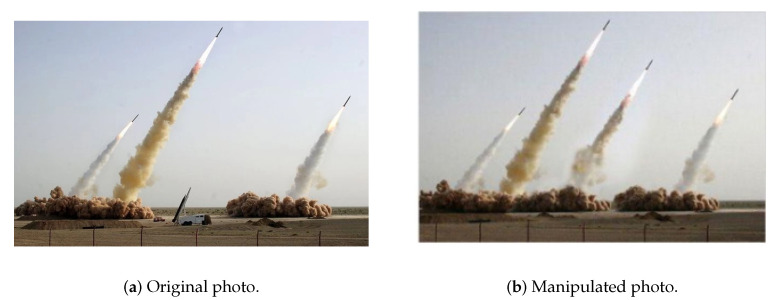
An example [24,25] of a copy–move manipulation technique.

There are two good examples of how copy–move manipulation can be used to great effect to spread fake information and wrest the original photo from its context. Nearly a decade ago, Iran was accused of doctoring a photo of its missile tests. The photo depicted in Figure 1 was released on Iran’s Revolutionary Guards official website, which claimed that four missiles were heading skyward simultaneously [22], when in fact only three missiles were launched. More recently, in July 2017, a fake image of Russian President Vladimir Putin was distributed on social media related to his meeting with US President Donald Trump during the 2017 G20 summit. This fake image garnered several thousand likes and retweets [23].

This manipulation technique consists of copying or moving parts of a photo to another place in the same photo. The goal is to give the illusion of having more elements in the photo than those that are really there. The main reason behind the increase of copy–move manipulation is the simplicity of the method. Textured areas such as grass, foliage, gravel, or fabric with irregular patterns, are ideal for this purpose because the copied areas will probably blend into the background and the human eye will not be able to easily discern any suspicious artifacts. As the copied parts came from the same digital photo, their noise, color palette, dynamic range, and other important properties will be compatible with the rest of the photo. This type of manipulation is difficult to detect by methods that look for statistical measurements’ incompatibilities in different regions of the photo [26].

**Figure 2:**
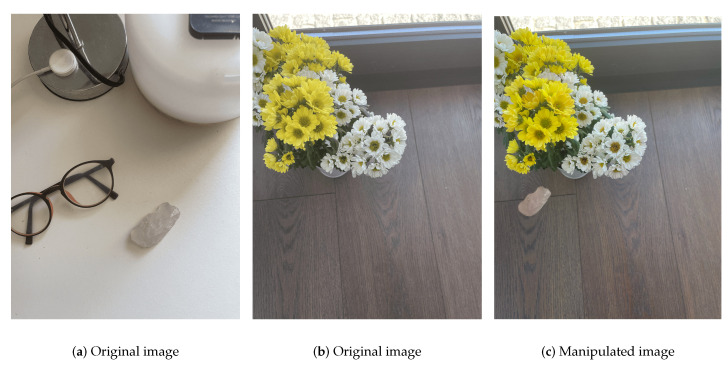
Splicing manipulation.

Splicing (Figure 2) consists of superimposing different regions of two photos, with deepfake being the most relevant consequence. It is an artificial and automated manipulation of media, usually made by employing AI techniques, in which a person’s face in an existing photo or video is swiped by someone else’s face. This manipulation is often used as an initial step of photo-montage, which is very popular in digital photo content editing. The splicing tampered image could be used in news reports, photography contests, key proof in academic papers, and so on, which could bring negative impacts.

As a result, it is an important issue to develop reliable splicing detection methods. In the forgery process, the manually introduced transitions of edges and corners are different from those in the legitimate photos. The differences are commonly described by the inconsistency and abnormality, and they are used for splicing detection [27].

**Figure 3:**
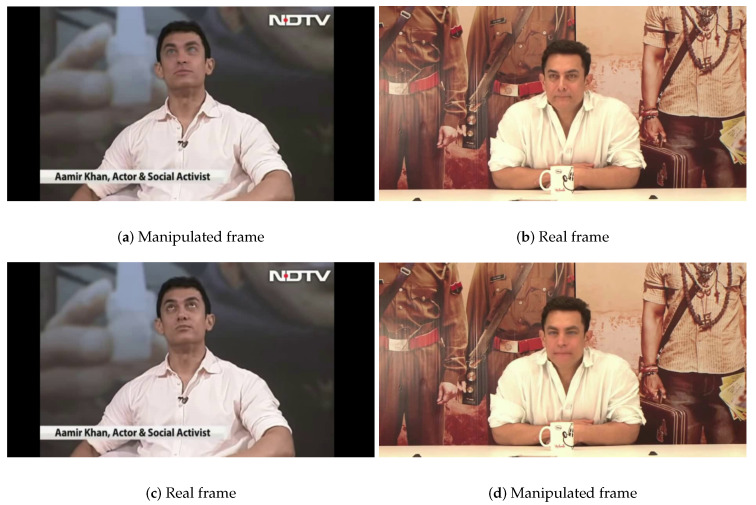
Comparison between fake and real frames in deepfake videos. These frames were extracted from videos in the Celeb-DFv1 dataset [21].

The term “deepfake” is the combination of “deep learning” and “fake” [9]. In general, deepfake is achieved by manipulating realistic videos with the aim to depict people saying or doing things that did not happen. This kind of manipulation is usually difficult to detect, as it uses real footage to make it the closest thing to reality. Figure 3 depicts two frames extracted from two videos, one being legitimate (Figure 3b,c) and the other manipulated (Figure 3a,d). As can be seen in Figure 3a in comparison with Figure 3c, the eyes seem a little foggy and are looking in opposite directions. If someone without knowledge of the real frame was looking at the manipulated one, they might think it was a cross-eyed person and not notice that it was a deepfake. In Figure 3d, similarly to Figure 3b, the face is a little foggy and even blurry, and the quality is not compatible with the rest of the photo. In some cases, when comparing two photos, it is easy to see which is the deepfake, but having access only to the manipulated image, with the naked eye, it is not so perceptible that the photo has been manipulated, further demonstrating the importance of creating modules to identify this type of manipulation.

While deepfake of photos and videos is not new and can be observed in a lot of digital content, it has leveraged powerful machine learning and AI techniques to improve content manipulation [28]. The most common ML methods used to improve deepfakes are based on deep learning and involve training generative neural network architectures, such as auto-encoders or Generative Adversarial Networks (GANs) [29]. By using GANs, two Artificial Neural Networks (ANN) work together to create a real-looking media. The first neural network, usually called “the generator”, tries to create new samples that are good enough to trick the second network training with a dataset containing real photos. In conclusion, a GAN can look at thousands of photos of a person and produce a new portrait that approximates those photos without being an exact copy of any one of them. Deepfake has garnered widespread attention, as it has been used in digital campaigns of spreading fake news. This manipulation technique is also responsible for digital kidnap, revenge porn, and financial fraud [30,31].

### 2.2. Techniques Used to Detect Multimedia Manipulation

Bearing in mind that the use of deepfake in digital crimes is a growing problem and has a great impact on today’s society, some algorithms were developed to tackle this type of manipulation, namely Difference of Gaussian (DoG) and Oriented Rotated Brief (ORB). DoG and ORB are two widely used techniques to automatically detect copy–move manipulation in photos. This method was suggested by Niyishaka et al. [32] and comprises three steps: corners detection with Sobel algorithm [33]; features extraction with DoG and ORB [32,34]; and, finally, features correspondence. This method combines detection techniques based on blocks and key points in a single model. A match is found between two points of interest if the distance is less than a predetermined threshold.

Unmasking deepfake with DFT and ML is a method described in [20]. It is based on a classical frequency domain analysis with DFT, followed by a classification based on ML techniques, namely by using Support Vector Machines (SVM). The frequency characteristics of a photo are analyzed in a space defined by a Fourier transform, namely by using a spectral decomposition of the input data, which indicates how the signal’s energy is distributed over a range of frequencies. Mathematically, DFT decomposes a discrete signal into sinusoidal components of various frequencies ranging from 0 (constant frequency, corresponding to the image mean value) up to the maximum of the admissible frequency, given by the spatial resolution. The frequency–domain representation of a signal carrying information about the signal’s amplitude and phase at each frequency is computed as described in (1):

(1) $$X_{k,l}=\sum_{n=0}^{N-1}\sum_{0}^{M-1}x_{n,m}\cdot e^{\left(-\frac{i2\pi }{N}k_{n}\right)}\cdot e^{\left(-\frac{-i2\pi }{M}l_{m}\right)}$$

**Figure 4:**
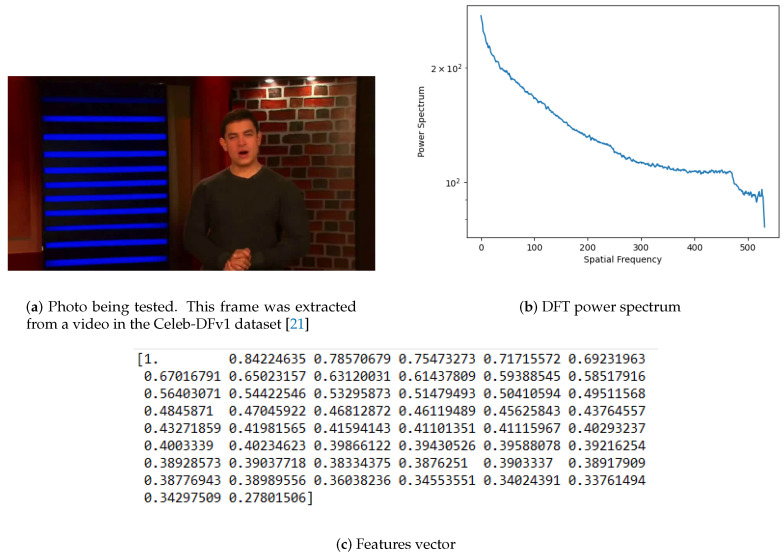
Photo features extraction by using Discrete Fourier Transform (DFT).

After applying a Fourier Transform to a photo, the returned values are represented in a new domain but within the same dimensionality. The output still contains 2D information, to which an azimuthal average is applied to compute a robust 1D representation of the DFT power spectrum. At this point, each frequency component is the radial average from the 2D spectrum previously calculated. In this case, the number of extracted features (frequency component) is a value chosen, taking into account computational time and classification scores. In the experiments made, fifty features were extracted from each photo. Figure 4b depicts the bidimensional representation of the DFT power spectrum of the photo depicted in Figure 4a. The resulting unidimensional array, containing the fifty selected features, is illustrated in Figure 4c.

After the features extraction, Support Vector Machines (SVM) is used to create a model based on a training dataset with manipulated and genuine photos. The model is then applied to a test dataset, to identify an optimal separating hyperplane that maximizes the margin between both classes (details described in Section 2.3).

Castillo and Yang [35] present a comprehensive review of the state-of-the-art deep learning-based methods for image forensics, both photos and videos. An exhaustive set of methods are introduced for a set of problems, namely median filtering, double JPEG, contrast enhancement, and general-purpose image processing operations. The performance obtained with the methods described, using distinct input features and datasets, is far above 90% accuracy.

One of the most impressive forms of ANN architecture is the Convolutional Neural Network (CNN) [36]. CNN is a deep learning algorithm which takes as input an input image, assigns importance (learning weights and biases) to various aspects and objects present in the image, and has the ability to differentiate one from the other. This type of neural network is comprised of neurons that self-optimize through learning. Each neuron receives an input and performs an operation, such as a scalar product followed by a nonlinear function [37].

The method described by Jafar et al. [38] applies a deep learning and CNN approach to detect a deepfake by using DFT in previously extracted mouth features (DFT-MF). Deepfake video extraction is completed by the moviePy tool and takes into account the occurrences of certain words. By using the identified face landmarks, the frames in which the person has his mouth closed are removed. In this method, a standard of two words per second and five words as a sentence indicator are defined. If the video has more than fifty fake frames, it is considered deepfake.

Recurrent Neural Networks (RNN) is a type of ANN that can have an internal memory to process sequences of inputs along the way in the net. This network allows previous outputs to be used as inputs while having hidden states. RNN models are mostly used in the fields of natural language processing and speech recognition [39]. CNN and RNN methods have been fully efficient to deal with the recognition of tampered images and videos [40]. Several authors have applied mixed CNN-RNN based architectures to detect anomalies in videos and recognize facial expression [41,42,43].

In short, the concept of a Generative Adversarial Network (GAN) is that two networks are trained to compete with one another. The “generator” network is trained to produce artificial photos that are indistinguishable from a given dataset of real photos, whereas the “discriminator” is trained to correctly classify all photos as being either real or coming from the generator (forged photos). In response to the development of GANs, the forensics investigators have begun to develop methods to detect whether or not a given image was generated by a network trained in a GAN framework [44].

Yang et al. [40] present an exhaustive survey of deep learning-based image forensics. A wide set of sources are presented, namely source camera identification, recaptured image forensic, computer graphics image forensics, GAN-generated image detection, and source social network identification. A vast number of detection methods were surveyed, each one using different network architectures and depth. The results are expressive and reveal the good performance obtained by deep learning-based approaches to tackle with image forensics.

In [45], a method is described to extract and analyze the similarity between audio (speech) and visual (face) modalities from within the same video. Effective cues corresponding to perceived emotion from the two modalities within a video are extracted and compared, to infer whether the input video is real or fake. To train the model, a real video is passed along with its deepfake through the network and obtains modality and perceived emotion embedding vectors for the face and speech of the subject. These embedding vectors are used to compute the triplet loss function to minimize the similarity between the modalities from the fake video and maximize the similarity between modalities for the real video. This method obtained an Area Under Curve (AUC) score of 84.4% on the DFDC dataset [46] and 96.6% on the DeepFake-TIMIT Dataset (https://www.idiap.ch/dataset/deepfaketimit (accessed on 22 June 2021)).

Despite the significant results attained with deep learning based methods, feature extraction and classifier methods, like DFT and SVM, respectively, have produced competitive results and can be well integrated into off-the-shelf forensic tools like Autopsy.

### 2.3. Support Vector Machines (SVM)

**Figure 5:**
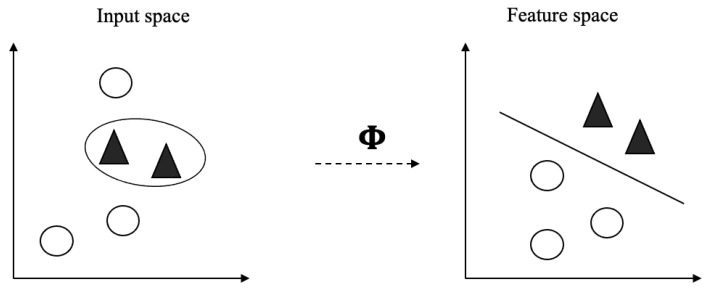
Mapping the training data nonlinearly into a higher-dimensional feature space, and constructing a linear separating hyperplane in the feature space.Linear separating hyperplane in the feature space.

SVM is a supervised learning classifier based on Vapnik’s Statistical Learning Theory and Structural Minimization Principle [47]. It is included in a set of kernel-based learning methods, in which the problem is addressed by mapping the data to a larger dimensional space. This mapping may not be linear, and the function that allows this mapping is called a kernel [48]. SVM introduced the concept of a kernel method for pattern analysis into machine learning scenarios [49], in which data are mapped into a high-dimensional feature space where each point represents a feature of the input data. This mapping is carried out by using a function ϕ (2), which is denominated by kernel function and where data are mapped into some feature space *F* via a nonlinear mapping, as depicted in Figure 5. Although it does not involve any computations in high-dimensional space, with the use of kernels, all computations needed are performed directly in input space [48].

(2) $$\Phi :R^{N}\to F$$

The linear hyperplane (in the feature space) that separates both classes is then selected. It only requires the evaluation of a kernel function and involves only the processing of dot products (3):

(3) k(x,y):=(Φ(x),Φ(y)).

Omitting details that can be found elsewhere [47], when using SVM for classification of data into two distinct classes, the overall idea is to find the optimal hyperplane between the positive and negative examples, which is defined as the one giving the maximum margin between the training examples that are closest to it. Figure 5 depicts the overall idea behind SVM. Support Vectors (SV) are the examples that lie closest to the separating hyperplane, and once this hyperplane is found, new examples can be classified simply by determining on which side of the hyperplane they fall. By using these support vectors, the margin of the classifier is maximized, and, by eliminating the support vectors, the position of the hyperplane changes.

SVM aims to maximize the margin between the data points and the hyperplane. The loss function that helps maximize the margin is hinge loss:

(4) c(x,y,f(x))=(1−y∗f(x))

The Regularization Parameter tells the SVM optimization how much it wants to avoid misclassification of each training example. If *C* is high, the optimization will choose a smaller margin hyperplane, so the misclassification rate of the training data will be low. In the opposite direction, if *C* is low, then the margin will be large, even if there are classification errors of the training data. The cost is 0 if the predicted value and the current value are of the same sign. If they are not, the loss value is calculated. A smoothing parameter is also added to the cost function, to balance margin maximization and loss. After adding the smoothing parameter, the cost function looks like (5):

(5) $$min_{w}{\lambda ||w||}^{2}+\sum_{i=1}^{n}\left(1-y_{i}<x_{i},w>\right)$$

A learning algorithm usually tries to learn the most common features (what differentiates one class from another) of a class and classification is based on the learned representative features (so classification is based on the differences between classes). SVM works the other way around, as it finds the most similar examples between classes, represented by the support vectors.

SVM models have been applied as learning classifiers to distinguish between original and tampered photos and videos. In [50], an SVM classifier is used to detect re-sampled images. The method is based on examining normalized energy density present within varying size windows in the second derivative of the frequency domain and exploits this characteristic mentioned to derive a 19-dimensional feature vector that is used to train the SVM classifiers. SVM is also used in the methods described previously in Section 2.2 [20,32].

## 3. Digital Forensics

Digital forensics has gained a growing interest in the criminal ecosystem (e.g., attorneys, prosecutors, trial, criminal police), as the number of cybercrimes and crimes using electronic equipment has increased in the past several years. Nowadays, the vast majority of crimes, ranging from the most traditional like murder or assault, to cybercrime, takes advantage of electronic devices connected to the Internet. This shift in the modus operandi has direct implications on the increasing number of equipment (e.g., PC, laptops, external storage devices, mobile phones, among others) seized by the police in the scope of a process, and consequently on the methodology adopted to analyze those devices.

Criminal police have been challenged to implement emergent methodologies to accelerate the analysis process, and, at the same time, to automatically extract, analyze, and preserve the digital evidence being collected in electronic equipment, namely disks, smartphones, and other devices with storage capacity. These tools embody techniques and procedures to produce a sustained reconstruction of events, to help digital forensics’ investigators build a list of evidence that may dictate information about the suspect’s innocence or guilt. The manual analysis by the criminal investigation team is still needed but oriented towards specific artifacts previously selected by the digital forensics tools and not necessarily in repetitive and tiresome tasks.

The protection of digital forensics information, and preservation of digital evidence, is achieved by establishing strict guidelines and procedures, namely detailed instructions about authorized rights to retrieve digital evidence, how to properly prepare systems for evidence retrieval, where to store any recovered evidence, and how to document these activities to guarantee data authenticity and integrity [51].

**Figure 6:**
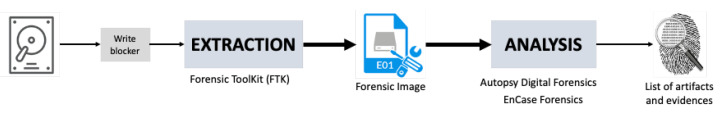
Overall procedure to extract and analyze electronic devices.

Figure 6 depicts the overall procedure to extract and analyze electronic devices, namely those with storage capabilities. The device is plugged into a write blocker, to prevent any write operation that may be done inadvertently. Then, by using a program to extract a raw image of the storage device, such as Forensic ToolKit (FTK, https://accessdata.com/ (accessed on 22 June 2021)), a E01 format image file is produced. Taking the E01 file as input, the digital forensics analysis starts, by using adequate tools, such as Autopsy Digital Forensics (https://www.autopsy.com/ (accessed on 22 June 2021)) or EnCase Forensic Software (https://security.opentext.com/encase-forensic (accessed on 22 June 2021)). The output produced is a list of artifacts that are worth investigating and which digital evidence has to be preserved to be accepted in court.

Autopsy is a widely used digital forensics tool to analyze a raw image file previously extracted from the electronic device. It is open-source and has distinct and well-appreciated visualization features to help the criminal investigator to assertively search the most relevant artifacts. Autopsy has the following key concepts:

- A case is defined in the Autopsy as a container with one or more data sources. Only one case can be opened at a time and is mandatory to start a digital forensics investigation in Autopsy.
- Data source is the term used to refer to raw disk images and logical files that are added to a case.
- Autopsy maintains a central SQlite or PostgreSQL database where all metadata files and results analysis are stored.
- After the data source analysis, the results are being gradually posted into a blackboard in the form of artifacts.

Data source analysis is made through available modules. The main reason to consider writing a module for Autopsy instead of a stand-alone tool is that Autopsy handles several data input types and ways to display the results to the user, which is an advantage as many forensic investigators do not have prior knowledge of programming [52].

Autopsy takes advantage of built-in modules and allows the community to develop new ones that may be based on already existing ones. These modules can be written in Java or Python (in this case, Autopsy uses Jython, to enable Python). There are four types of modules in Autopsy, namely ingest, report, content viewers, and result viewers.

**Figure 7:**
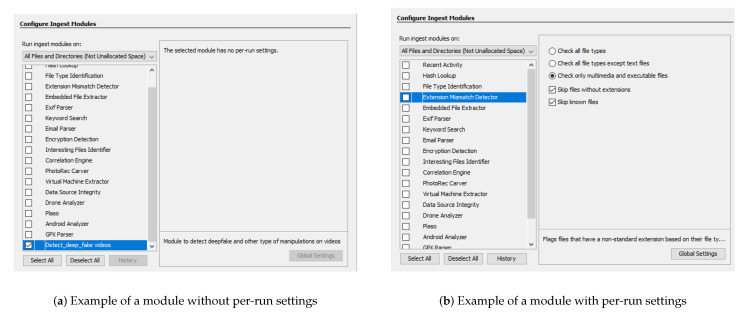
List of modules available in the Autopsy tool.

Ingest modules, depicted in Figure 7, are executed when a new data source is added to a case. They are called “File Ingest Modules” and are executed to examine the contents of a group of files in the data source. For example, “Data Source Ingest” modules are executed once for each image or set of logical files, to analyze artifacts.

Report modules are typically executed after a user has examined the results. The purpose of this module is to execute analysis and report the results obtained. Content viewers are graphical and focus on displaying a file in a specific form. They can be used, for example, to view a file in hexadecimal format. Finally, result viewers show information about a set of files. They are used, for example, to view a set of files in a table.

Some modules like File Type Identification, Email Parser, and Encryption Detection are available through Autopsy as illustrated in Figure 7. For example, the File Type Identification module identifies files based on their internal signatures and does not rely on file extensions. The Email Parser module identifies MBOX, EML, and PST format files based on file signatures, extracting the e-mails from them, and adding the results to the blackboard artifact for each message. The Encryption Detection module searches for files that could be encrypted using both a general entropy calculation and more specialized tests for certain file types. It is also possible to use modules developed by the third-parties. In [53], the authors present a module to successfully recover messages exchanged between TikTok users through the app communication channels. It is also possible to obtain the list of TikTok contacts of a user’s account, photos linked to the app, and TikTok videos watched by the user’s smartphone. Another example is described in the module described in [54], which allows forensic investigators to collect the needed information about Cortana, the new voice-activated personal digital assistant of the Windows 10 operating system.

XRY mobile forensics tool from MSAB (https://www.msab.com/products/xry/ (accessed on 22 June 2021)) is an intuitive and efficient software for Windows. It allows a fast and secure high-quality data extraction from mobile devices while maintaining the integrity of the evidence. XRY allows a rapid logical and physical extraction of files, and its file format maintains secure and accountable evidence at all times, with complete forensic auditing and protection of evidence from the moment the extraction begins.

Cellebrite (https://www.cellebrite.com/en/home/ (accessed on 22 June 2021)) is an Israeli toolset used for the collection, analysis, and management of digital data. It is a competitor of XRY for mobile device extraction and analysis, providing a wide set of features to extract data from digital devices.

EnCase from Opentext Security (https://security.opentext.com/encase-forensic (accessed on 22 June 2021)) has several products designed for forensics, security analytic, and e-discovery use. Encase is traditionally used in forensics to recover evidence from seized hard drives and support mobile devices’ extraction and analysis. It allows the investigator to conduct an in-depth analysis of user files to collect evidence such as documents, pictures, Internet history, and Windows Registry information, among other features.

Forensic ToolKit (FTK) from AccessData (https://accessdata.com/products-services/forensic-toolkit-ftk (accessed on 22 June 2021)) is an open-source tool that provides real-world capabilities that help forensics’ digital investigation teams separate critical data from trivial details and protect digital information while complying with digital regulations. This tool can be used by both criminal police and the private sector to perform complete forensic examinations of a computer. It includes customizable filters that allow the examiner to inspect thousands of files, including locating emails purportedly excluded from a computer; this feature is compatible with Outlook, AOL, Outlook Express, Netscape, Earthlink, Yahoo, Hotmail, Eudora, and MSN email.

The use of digital forensics tools is crucial to automate the extraction and analysis of electronic devices in the context of digital forensics. Autopsy has been widely used in forensics analysis and third-party modules have a positive impact on implementing additional and specific features. In the scope of this paper, two ingest modules were developed to detect deepfake digital photos and videos, respectively. The modules are described in Section 4 and are ready to be incorporated into the Autopsy forensics tool.

## 4. Architecture

This section describes the architecture that was deployed to process input videos and to classify them as being genuine or manipulated. It also describes the Autopsy module developed to classify videos in a digital forensics context and the dataset created for this context.

### 4.1. General Architecture

**Figure 8:**
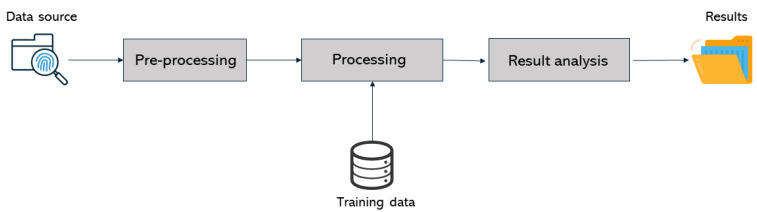
Overall architecture of the standalone application and Autopsy modules.

The overall architecture of the standalone application developed to classify photos and videos is depicted in Figure 8. It has three main building blocks: pre-processing, processing, and results analysis.

To obtain a functional deepfake detection system using Discrete Fourier Transform and Machine Learning, it is necessary for a first step to obtain the input data to feed the classification model, which will be used to classify each image as manipulated (deepfake) or legitimate.

**Figure 9:**
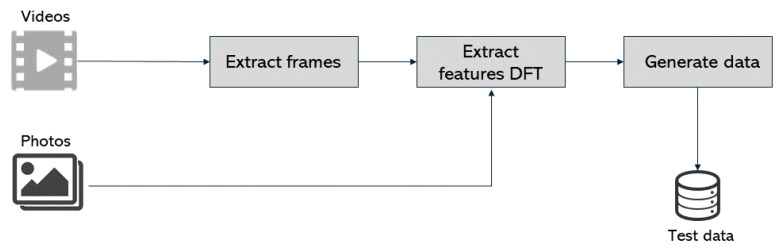
Pre-processing phase.

Pre-processing is depicted in Figure 9 and consists initially of taking three to four frames per second from the input videos. This was achieved by creating a Python script, and all the frames extracted are added to the final dataset. By having all the photos in the dataset, the features’ extraction is made by applying the DFT method described in Section 2.2 [20]. The output is a labeled input datasets for both training and testing. The preprocessing phase reads the photos through the OpenCV library and further extracts their features [20]. Using this method, exactly fifty features were obtained for each photo that were then loaded into a new file with the corresponding label (0 for fake photos and 1 for the genuine ones). At the end of the preprocessing phase, a fully labeled dataset is available and ready to feed the SVM model.

**Figure 10:**
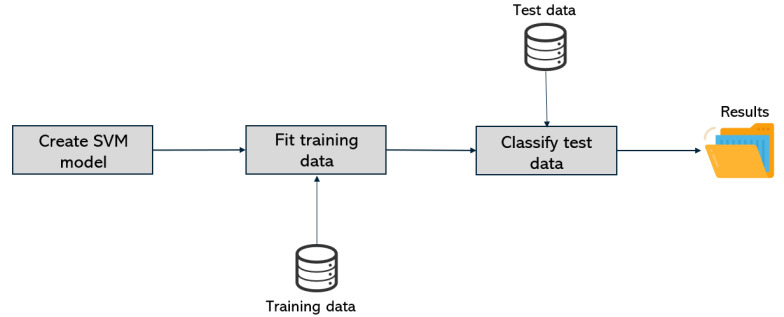
Processing phase.

The processing phase, depicted in Figure 10, corresponds to the SVM processing. In a first step, the following parameters were chosen: the RBF (Radial basis function) kernel and a regularization parameter of 6.37. This choice took into account the best practices adopted for similar experiments and the comparison with other parameters.

**Figure 11:**
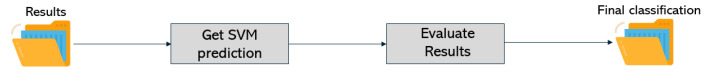
Results analysis phase.

The implementation of SVM processing was made through the scikit-learn library for Python 3.9. With the generated data ready to be classified and the SVM model created, the results analysis phase follows and is depicted in Figure 11.

The model created by SVM at the processing phase is then used to get a prediction for each photo in the testing dataset. The tests were carried out with a 5-fold cross-validation, by splitting the dataset into ten equal parts and using nine for training and one for testing. The dataset is balanced, regarding the number of fake and genuine photos and videos.

For each SVM model evaluation, the results obtained include the confusion matrix, precision, recall and F1-score; and the calculated prediction that allows us to deduce the probability of an image has been manipulated.

### 4.2. Autopsy Module Architecture

As stated before, Autopsy is among the most used digital forensics applications and is open to the integration of third-party modules. Autopsy processes the input data and shows the results by using report modules.

Autopsy uses Jython in new modules development, to enable Python scripting. Jython is converted into Java byte code and runs on the JVM. As it is limited to Python 2.7, to overcome this limitation and the fact that some libraries used by the SVM classification method did not work with Python 2.7, three Python executables were created: one to extract frames from videos; another to process photo’s features; and the third one to create the SVM model and to classify the photos.

**Figure 12:**
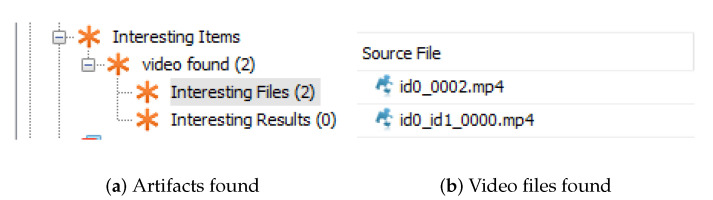
Videos found in data source—Autopsy module.

The data source ingest module that runs against a data source added to the Autopsy, was developed, and its architecture is similar to Figure 12. To start this analysis, it is necessary to create a new “case” inside Autopsy and add one data source to it. An example of a data source is a disk image. Then, the module starts by extracting each video within the data source added to the Autopsy case and saves them in a temporary directory. Only videos with the extension “.mp4” were considered in the processing.

For each video stored in the temporary directory, the first script is performed where three to four frames per second (depending on the original number of frames per second and the video duration) will be extracted and saved. The second executable then extracts the features from each frame stored and outputs obtained and, with the training file already created and distributed with the module, feeds the last Python executable, which creates the SVM classifier. The artifacts with the classification results are calculated and posted in the Autopsy blackboard, which are further displayed to the user.

**Figure 13:**
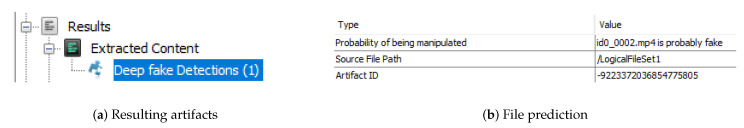
Artifacts with final classification—Autopsy module.

The model outputs a prediction of fakeness, as depicted in Figure 13. In the case of classifying if a video is manipulated or not, if a third or more of the frames of a video are classified as fake, it is considered that it is likely to be deepfake.

The standalone application architecture matches the Autopsy data source ingest module (Figure 7). The standalone application was developed before the Autopsy module, which gave the possibility to develop and test the method while disregarding the needed compatibility with the Python libraries and with the strict format that is required by Autopsy for the development of new modules.

## 5. Datasets

A dataset containing both people’s faces and objects was created to train and test the SVM-based classification model. The dataset used in [20] is a compilation of photos available in the CelebA-HQ dataset [55], Flickr-Faces-HQ dataset [56], “100 K Faces project” (https://generated.photos/ (accessed on 22 June 2021)) and “this person does not exist” project (https://thispersondoesnotexist.com/(accessed on 22 June 2021)). Table 1 itemizes the datasets collected and used in the experiments.

Some complexity was added to the dataset, by including objects and others people’s faces, being possible to detect other types of manipulations aside from deepfake. The COVERAGE dataset [57] is a copy–move forgery database with similar but genuine objects that contains 97 legitimate photos and 97 manipulated ones. The Columbia Uncompressed Image Splicing Detection Evaluation Dataset [58] was also added, which consists of high-resolution images, 183 authentic (taken using just one camera and not manipulated), and 180 spliced photos. An additional 14 legitimate and 14 fake ad hoc photos were also added, containing splicing and copy–move manipulations. For the video, Celeb-DF [21] was used to provide fake and real videos to train the model. This dataset contains 795 fake videos and 158 real ones extracted from Youtube. To combine these videos with the rest of the dataset, three frames per second were extracted from each video being treated as a photo thenceforth. In total, 6201 frames were extracted from real videos and 31,551 from fake ones.

To use these photos to train and test our model, the dataset must be balanced. To achieve that, if at some point we have more real photos than fake ones, we only use the minimum between them. To be more specific, as we have 31,551 fake photos extracted from videos and 6201 real photos, we will only use 6201 photos from the fake ones, with 12,402 photos extracted from videos in total. Adding up all datasets containing only photos, we have 20,291 fake photos and 20,294 real ones. Putting it all together, the new dataset used in this paper is balanced and has 52,990 photos divided into two classes: 26,495 genuine (or real) photos and 26,495 that were manipulated. Table 2 specifies the composition of the datasets tested, namely for photos and videos. For each dataset, the number of examples used for training and testing is also indicated. The results presented in Section 6 were validated through a 5-fold cross-validation methodology. That is, each dataset was equally divided into five parts, each one being tested against the model trained with the remaining four parts.

The Autopsy modules are optimized for Autopsy version 4.15.0 and were developed in Python version 3.9. The experiments were carried on in a PC with Windows 10, 8 GB RAM and AMD Ryzen 5 2600.

## 6. Results Analysis

This section describes the results obtained from the experiments and the corresponding analysis. The experiments were validated by a 5-fold cross-validation approach, for the dataset created described in Section 5. Evaluation metrics are described in Section 6.1, and the analysis of the results is presented in Section 6.2. Finally, Section 6.3 describes the results obtained with the benchmark of DFT-SVM and a CNN-based model.

### 6.1. Evaluation Metrics

The metrics used to evaluate the results were Precision (P), Recall (R), and F1-score, which can be calculated through the well-known and documented confusion matrix depicted in Table 3 [59].

In the confusion matrix, each row represents the instances in a predicted class, while each column represents the instances in an actual class. The positive class refers to the manipulated photos, while the negative class represents the original and unmanipulated ones. True Positives (TP) represent the events where the model has correctly predicted the positive class, while True Negatives (TN) are the events correctly predicted as negative, that is, genuine photos. False Positives (FP) and False Negatives (FN) evaluate the events that were incorrectly predicted by the model, namely those that correspond to legitimate photos classified as manipulated and those manipulated that were classified as genuine, respectively.

Precision and Recall correlate the metrics described above. Precision measures the percentage of examples identified as true that are genuine and correspond to real photos or videos. Precision is calculated by (6):

(6) $$P=\frac{TP}{\left(TP+FP\right)}$$

Recall is the percentage of manipulated images that we could find of the total number of manipulated images. Recall corresponds to the following (7):

(7) $$R=\frac{TP}{\left(TP+FN\right)}$$

F1-score is a harmonic mean between Precision and Recall. The range for F1-score is between [0, 1] and measures the preciseness and robustness of the classifier—that is, the number of instances that were correctly classified and those that were misclassified, respectively. F1 measure is calculated by (8):

(8) $$F1=2*\frac{P*R}{\left(P+R\right)}$$

Accuracy (9) is calculated by the ratio between the correctly classified examples (real and fake photos and videos) and the total number of examples (correctly and incorrectly classified):

(9) $$A=\frac{TP+TN}{\left(TP+TN+FP+FN\right)}$$

### 6.2. Results with DFT-SVM

Table 4 describes the results obtained with a 5-fold cross-validation to the dataset of digital photos. The table highlights the partial results obtained in each split, namely the number of FP, FN, TP, and TP, as well as the calculated values for P, R, F1, and accuracy. The corresponding mean scores obtained with the 5-fold cross-validation are also indicated.

The mean value obtained for accuracy (*A*) is 99.51%, which surpasses the result of 93.52% achieved in [32]. The number of incorrectly classified examples, namely false positives and false negatives, is low, having a mean value of 14 and 25.8, respectively.

The results attained with a 5-fold cross-validation processing for the dataset of videos are presented in Table 5. The mean values for F1-score and accuracy are 79.55% and 77.94%, respectively. Regarding misclassified examples, the average values for FP and FN are, respectively, 365 and 180 for a total amount of 2480 examples.

Considering that videos are composed of a set of photos, a third experiment was made to accommodate both multimedia content types. Table 6 presents the results obtained with the whole dataset composed of 52,990 examples, applying a 5-fold cross-validation.

It is possible to observe that the mean values for precision, recall, and F1-score are respectively 81.14%, 98.79%, and 89.10%. The calculated mean accuracy is 87.92%, and the overall results outperform those attained and documented in [20]. We can conclude that we obtained very satisfactory results considering the work already developed and available in the literature for SVM-based methods, despite using a dataset with more diversity on manipulation types. The results could be even better with an even more diverse training dataset and preserving the same quality of photos.

**Figure 14:**
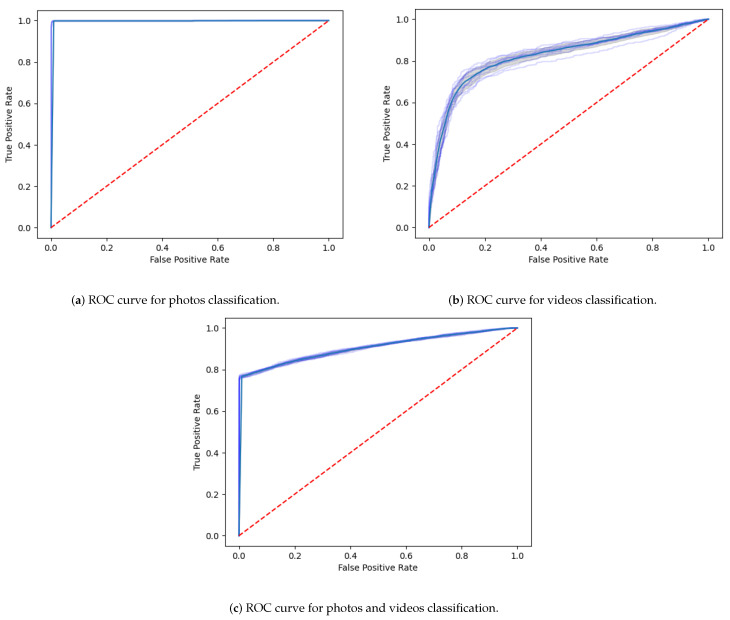
Receiver Operating Characteristic (ROC) curves calculated for photos and videos processing.

The Receiver Operating Characteristic (ROC) curve is a versatile and well-adopted technique to graphically show the performance of a binary classifier. It plots the probability of detection (TPR) versus the probability that a false alarm (FPR) may happen, at different classification thresholds. Figure 14a depicts the ROC curve for video classification, where it is possible to observe its high performance, as the fake videos classifier gives a curve closer to the top-left corner.

Figure 14b illustrates the ROC curve related to the processing of the whole dataset, with photos and videos. It is also possible to observe the good performance of the classifier, in which the curve is pushed to the upper left corner of the graphic.

### 6.3. Benchmark with CNN-Based Methods

The SVM method proposed in this paper was compared with a CNN-based method, to benchmark both methods in terms of classification performance and processing time. CNNs are comprised of three types of layers: convolutional layers, pooling layers, and fully-connected layer [37]. The CNN created to benchmark with an SVM model consists of a convolutional layer with relu, following a pooling layer, another convolutional layer with relu, another pooling layer followed by a flatten layer to pass the multidimensional input to one dimension, and ending with two dense layers (fully-connected layers), one with relu and one with softmax. The CCN architecture was built on the top of Tensorflow (https://www.tensorflow.org/ (accessed on 22 June 2021)), being a neural network library Keras used to create the training and testing datasets.

Table 7 depicts the results obtained with the benchmarking of the videos dataset processing, namely the comparison of the results obtained with the DFT-SVM and CNN based methods.

Regarding videos processing, when comparing with previously documented experiments, it is possible to note that, using the Celeb-DF dataset as part of the input dataset, the results outperform those obtained by [38], which uses DFT with Mouth Features (MF) to extract features and a CNN-based method to classify the videos. The reported accuracy was 71.25%.

Table 8 describes the results obtained with the benchmarking of the photos dataset processing, namely the comparison of the results obtained with the DFT-SVM and CNN based methods. It is possible to observe that the results obtained are similar to those reported by the authors in [20], by applying the same DFT-SVM method, but with a restricted dataset.

Regarding the processing times, CNN and SVM based methods possess quite distinct realities. Table 9 depicts the observed time spent by SVM and CNN based methods, under the same hardware setup, for processing the aggregated dataset proposed in Section 5. The values indicated in Table 9 are only related to the testing processing time, and do not include the preprocessing phase and features extraction. It is possible to observe that the processing time consumed by the CNN-based model is considerably higher than that spent by the SVM-based method, for both photo and video processing.

Deep learning based methods have been widely used, and are considered state-of-the-art cutting edge in what image and video forensics are about [35,40]. However, the features extraction methods and the overall functioning of deep learning based models, such as CNN and RNN, are time-consuming to process, and less flexible to be embedded into standalone off-the-shelf digital forensics tools, like Autopsy. Regarding the DFT-SVM compound method, the results achieved with the dataset proposed in this paper are competitive with the CNN model for both photos and videos, with a significantly lower processing time. The trade-off between the processing time and the evaluation performance obtained by DFT-SVM method [20] should thus be taken in account in the development of forensic tools to support and help criminal investigator’s digital forensics daily routine.

By observing the available Autopsy third-party modules listing (https://github.com/sleuthkit/autopsy_addon_modules (accessed on 22 June 2021)), and also the modules developed by the Autopsy’s community (https://www.osdfcon.org/ (accessed on 22 June 2021)), to the best of the authors’ knowledge, there is not yet a registered and ready-to-use Autopsy module designed and developed to detect deepfake and digitally manipulated photos and videos in a forensics context.

## 7. Conclusions and Future Work

This paper described the development of an application to detect tampered multimedia content. An SVM-based method was implemented in a standalone application, to process the previously extracted features obtained by a DFT calculation in each multimedia file. Two modules for Autopsy digital forensics tool were developed, namely a module to detect tampered photos and another one to identify deepfake videos. The fundamentals behind digital forensics, SVM, and DFT were described. The most relevant and up-to-date literature review related to digital forensics on multimedia content was made, namely the survey on deep learning-based methods applied to photos and videos forensics.

The deliverables obtained with this research, namely the ready-to-use Autopsy modules, give a helping hand to digital forensics investigators and leverage the use of ML techniques to fight cybercrime activities that involve multimedia files. The overall architecture and development take advantage of two well-known and documented techniques to deal with feature extraction in multimedia content and to automatically detect from learning classifier models, respectively, the Discrete Fourier Transform (DFT) technique to extract features from photos, and SVM to classify files. Both techniques were incorporated in the developed standalone application, which was further integrated as two separated Autopsy modules. The dataset proposed in [20] was extended with different sources, mainly to accommodate deepfake videos. The final dataset has about 53,000 photos, enriched with faces and objects, where it is possible to find examples of deepfake, splicing, and copy–move manipulations. Some of the photos are frames extracted from deepfake videos.

The results were presented in three distinct dimensions: the classification performance obtained with a 5-fold cross-validation for photos and videos processing; the benchmark between SVM and CNN-based methods using the dataset proposed in this paper; and the processing time of SVM and CNN-based methods. The results obtained with SVM were promising and in line with previous ones documented in the literature for the same method [20]. It was possible to achieve a mean F1-score of around 99.5% for manipulated photos detection and 78.4% for deepfake video detection. Deep learning methods, namely CNN-based, outperformed those achieved by SVM, however with a considerably higher processing time. Strictly concerned with daily-routine digital forensic interest, despite the better results obtained with CNN-based methods, the trade-off with the processing time benefits the use of the SVM method with the features extracted by DFT.

By analyzing the misclassified photos and video frames, a possible cause could be related to the low resolution of the photos. A richer dataset with heterogeneous examples regarding the resolution of the photos would improve the overall results obtained. The optimal number of features that should be extracted from the photos, and its impact in computational time, is also worth investigating. An ensemble of learning classifiers, composed of both deep learning and SVM based methods, could benefit both the performance obtained and the processing time. A net model for forensic detection using CNN, eventually using a different architecture, is also worth investigating and implementing.

Besides the well-accepted implementation in Autopsy modules, an emergent subject that may benefit from the developed architecture is the detection of fake news and the spread of hate speech in social networks. The low processing time and the high performance obtained with the DFT-SVM method make it eligible to be incorporated as a plugin that may be used easily, and in real time, to detect the fakeness level of multimedia content spread in social networks.

## Figures and Tables

**Table 1:** Composition of the dataset.

Name	Fake	Real
CelebA-HQ dataset	-	10,000
Flickr-Faces-HQ dataset	-	10,000
“100 K Facesproject”	10,000	-
“this person does not exist”	10,000	-
COVERAGE dataset	97	97
Columbia Image Splicing Dataset	180	183
Created by us	14	14
Celeb-DFv1	795	158
	21,086	20,452

**Table 2:** Composition of the training and testing datasets.

	Training	Testing
Photos	32,464	8116
Videos	9920	2480
Photos and Videos	42,384	10,596

**Table 3:** Confusion matrix.

	Positive	Negative
**Positive**	TP	FP
**Negative**	FN	TN

**Table 4:** Results obtained with 5-fold cross-validation against the dataset of photos.

	TP	TN	FP	FN	Precision	Recall	F1-Score	Accuracy
Split 1	3999	4070	20	27	0.9950	0.9933	0.99941	0.9942
Split 2	4089	3986	17	24	0.9958	0.9942	0.9950	0.9949
Split 3	4091	3983	11	31	0.9973	0.9925	0.9949	0.9948
Split 4	3972	4111	11	22	0.9972	0.9945	0.9959	0.9960
Split 5	4010	4070	11	25	0.9973	0.9938	0.9955	0.9952
Mean	4032.2	4044	14	25.8	0.9965	0.9941	0.9953	0.9951

**Table 5:** Results obtained with 5-fold cross-validation against the dataset of videos.

	TP	TN	FP	FN	Precision	Recall	F1-Score	Accuracy
Split 1	1092	891	307	190	0.7746	0.8474	0.8093	0.7996
Split 2	1054	862	385	179	0.7324	0.8548	0.7889	0.7726
Split 3	1066	883	351	180	0.7523	0.8555	0.8006	0.7859
Split 4	1033	884	385	178	0.7285	0.8530	0.7858	0.7730
Split 5	1055	855	397	173	0.7266	0.8591	0.7873	0.7702
Mean	1060	865	365	180	0.7438	0.8548	0.7955	0.7794

**Table 6:** Results obtained with 5-fold cross-validation against the dataset of photos and videos.

	TP	TN	FP	FN	Precision	Recall	F1-Score	Accuracy
Split 1	5319	4016	1192	71	0.8169	0.9868	0.8939	0.8808
Split 2	5216	4104	1221	57	0.8103	0.9992	0.8909	0.8974
Split 3	5228	4055	1248	67	0.8073	0.9873	0.8883	0.8770
Split 4	5201	4137	1203	57	0.8121	0.9891	0.8819	0.8815
Split 5	5211	4101	1218	68	0.8105	0.9871	0.8902	0.8787
Mean	5235	4082.6	1216.4	64	0.8114	0.9879	0.8910	0.8792

**Table 7:** Benchmark videos.

	Precision	Recall	F1-Score	Accuracy
DFT with SVM	0.7438	0.8548	0.7955	0.7794
CNN	0.8820	0.8045	0.8415	0.8387

**Table 8:** Benchmark photos.

	Precision	Recall	F1-score	Accuracy
DFT with SVM	0.9965	0.9941	0.9953	0.9951
CNN	0.9970	0.9966	0.9968	0.9967

**Table 9:** Processing time spent for videos and photos, in the format hh:mm:ss.

	Photos	Videos
DFT with SVM	00:00:51	00:02:00
CNN	06:36:00	02:40:00

## Data Availability

Data is publicly available under an MIT license, at the following GitHub repository: https://github.com/saraferreirascf/Photo-and-video-manipulations-detector.

## Abbreviations

The following abbreviations are used in this manuscript:

A	Accuracy
AI	Artificial Intelligence
ANN	Artificial Neural Networks
AUC	Area Under Curve
CNN	Convolutional Neural Networks
DFT	Discrete Fourier Transformation
DoG	Difference of Gaussian
FTK	Forensic Tool Kit
FN	False Negative
FP	False Positive
GAN	Generative Adversarial Network
ML	Machine Learning
ORB	Oriented Rotated Brief
PST	Personal Storage Table
P	Precision
R	Recall
RNN	Recurrent Neural Network
ROC	Receiver Operating Characteristic
SV	Support Vector
SVM	Support Vector Machines
TN	True Negative
TP	True Positive
